# The complete chloroplast genome sequence of *Ziziphus incurva*

**DOI:** 10.1080/23802359.2019.1674706

**Published:** 2019-10-11

**Authors:** Yi Wang, Jiabo Hao, Xiaolong Yuan, Bin Lu

**Affiliations:** Laboratory of Forest Plant Cultivation and Utilization, Yunnan Academy of Forestry, Kunming, Yunnan, People's Republic of China

**Keywords:** *Ziziphus incurva*, chloroplast, Illumina sequencing, phylogenetic analysis

## Abstract

The first complete chloroplast genome sequences of *Ziziphus incurva* were reported in this study. The complete chloroplast genome (cpDNA) sequence of *Z. incurva* was determined from Illumina HiSeq pair-end sequencing data. The cpDNA is 160,920 bp in length, contains a large single-copy region (LSC) of 88,778 bp and a small single-copy region (SSC) of 19,172 bp, which were separated by a pair of inverted repeat (IR) regions of 26,477 bp. The genome contains 129 genes, including 84 protein-coding genes, 8 ribosomal RNA genes, and 37 transfer RNA genes. The overall GC content of the whole genome is 36.8% and the corresponding values of the LSC, SSC, and IR regions are 34.6, 30.8, and 42.7%, respectively. Further, the phylogenomic analysis showed that *Z. incurva* clustered together with *Z. jujube*, *Z. mauritiana, and Z. spina-christi*.

Ziziphus is one of the most economic genera among Rhamnaceae, and some authors use Zizyphus as the spelling for the genus name (Paclt 1999). Ziziphus is widespread in tropical, subtropical, and temperate areas of both hemispheres and it has approximately 170 species in the world. And 18 species of Ziziphus are cultured in China (Li et al. 2010). *Ziziphus incurva* is the species of the genus Ziziphus within the family Rhamnaceae, is distributed in southern China, India, Nepal, and Bhutan. *Ziziphus incurva* is widely used as folk medicine in Nepal (Devkota et al. 2013). *Z. incurva* also is important tropical and subtropical cultivation of fruit trees in India and Taiwan, China (Ni et al. 2011). However, there has been no genomic studies on *Z. incurva*.

Herein, we reported and characterized the complete *Z. incurva* plastid genome (MN017132). One *Z. incurva* individual (specimen number: 5309270745) was collected from Lincang, Yunnan Province of China (23°13′6″ N, 99°20′27″ E). The specimen is stored at Yunnan Academy of Forestry Herbarium, Kunming, China, and the accession number is YAFH0012755. DNA was extracted from its fresh leaves using DNA Plantzol Reagent (Invitrogen, Carlsbad, CA, USA).

Paired-end reads were sequenced by using Illumina HiSeq system (Illumina, San Diego, CA, USA). In total, about 28.1 million high-quality clean reads were generated with adaptors trimmed. Aligning, assembly, and annotation were conducted by CLC de novo assembler (CLC Bio, Aarhus, Denmark), BLAST, GeSeq (Tillich et al. 2017), and GENEIOUS v 11.0.5 (Biomatters Ltd, Auckland, New Zealand). To confirm the phylogenetic position of *Z. incurva*, other six species of family *Rhamnaceae* from NCBI were aligned using MAFFT v.7 (Katoh and Standley 2013) and maximum likelihood (ML) bootstrap analysis was conducted using RAxML (Stamatakis 2006); bootstrap probability values were calculated from 1000 replicates. *Hippophae rhamnoides* (KY794808) was served as the out-group.

The complete *Z. incurva* plastid genome is a circular DNA molecule with the length of 160,920 bp, contains a large single-copy region (LSC) of 88,778 bp and a small single-copy region (SSC) of 19,172 bp, which were separated by a pair of inverted repeat (IR) regions of 26,477 bp. The overall GC content of the whole genome is 3 36.8%, and the corresponding values of the LSC, SSC, and IR regions are 34.6, 30.8, and 42.7%, respectively. The plastid genome contained 129 genes, including 84 protein-coding genes, 8 ribosomal RNA genes, and 37 transfer RNA genes. Phylogenetic analysis showed that *Z. incurva* clustered together with *Z. jujube, Z. mauritiana, and Z. spina-christi* (Figure 1). The determination of the complete plastid genome sequences provided new molecular data to illuminate the *Rhamnaceae* evolution.

**Figure 1.**
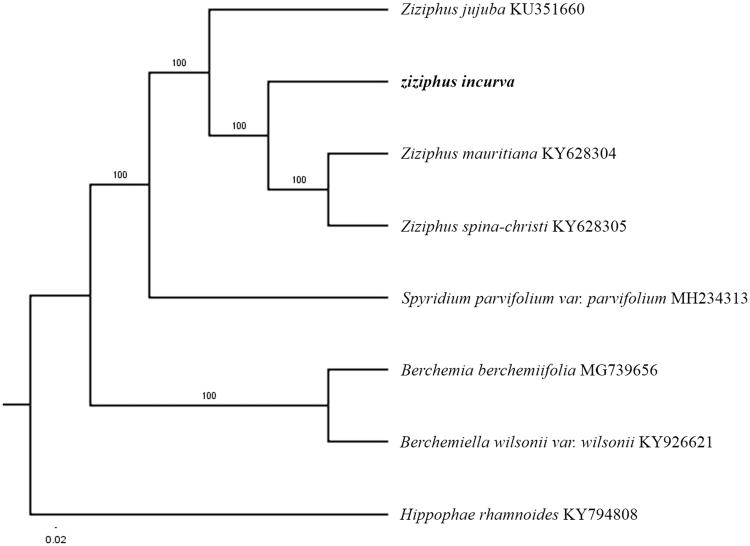
The maximum-likelihood tree based on the 7 chloroplast genomes of *Rhamnaceae*. The bootstrap value based on 1000 replicates is shown on each node.
